# Comparative analysis of lifestyle behaviors and dietary intake among obese and non-obese individuals following bariatric surgery: a secondary data analysis from 2020 to 2022

**DOI:** 10.3389/fnut.2023.1273164

**Published:** 2023-10-30

**Authors:** Nora A. Althumiri, Nasser F. Bindhim, Abdulaziz E. Aldabaeab, Norah AlMousa, Rugayah Aljabbary, Arwa Alumran

**Affiliations:** ^1^Informed Decision Making (IDM), Riyadh, Saudi Arabia; ^2^Sharik Association for Research and Studies, Riyadh, Saudi Arabia; ^3^College of Medicine, Alafia University, Riyadh, Saudi Arabia; ^4^King Fahad Hospital of the Imam Abdulrahman Bin Faisal University, Dammam, Saudi Arabia; ^5^Health Information Management and Technology Department, College of Public Health, Imam Abdulrahman Bin Faisal University, Dammam, Saudi Arabia

**Keywords:** lifestyle behaviors, bariatric surgery, post bariatric surgery, Saudi Arabia, dietary intake

## Abstract

**Objective:**

The aim of this research is to perform a comparative examination of lifestyle habits and dietary consumption between obese and non-obese subjects who have undergone bariatric surgery. This is done with the intent of investigating the disparities in obesity outcomes attributable to these elements.

**Method:**

This study involves a secondary analysis of cross-sectional data obtained from the Sharik Diet and Health National Survey (SDHNS). To ensure a representative distribution of participants, the SDHNS employs a proportional quota sampling strategy, with stratification based on age, sex, and geographic location within Saudi Arabia’s 13 administrative regions, utilizing the ZDataCloud® system for this purpose. The data, collected between 2020 and 2022 from over 15,000 participants, were screened to identify the eligible records of individuals who underwent bariatric surgery.

**Results:**

Within the entire sample, a mere 5.0% (806 individuals) had undertook bariatric surgery, with females comprising 54% of this specific subgroup. The average age within this group was 38.85 years (SD 13.02) and range (18–87). Post-operative results showed that 33% of these individuals remained classified as obese. Utilizing the backward likelihood ratio regression model, it was determined that factors including age, decreased consumption of fresh juices and chicken, as well as current tobacco use, were significantly associated with persistent obesity.

**Conclusion:**

The findings of this study suggest an association between the non-obese group and healthier lifestyle choices, including the consumption of high-protein diets and fresh juices, alongside a decreased prevalence of smoking. These observations underscore the significance of maintaining a healthy lifestyle for positive weight loss outcomes following bariatric surgery.

## 1. Introduction

Obesity is an escalating public health issue, with complications that substantially reduce quality of life and life expectancy (1). In 2020, Saudi Arabia’s a national study showed that obesity rate (for those with a BMI ≥ 30 kg/m^2^) reached 21.7% (2). Among the Saudi population, obesity has a significant correlation with conditions such as Type 2 Diabetes Mellitus (T2DM), hypercholesterolemia, hypertension, lung diseases, rheumatoid arthritis, sleep apnea, colon diseases, stroke and heart diseases, and thyroid disorders (2). The cost of treating obesity in Saudi Arabia is currently about 2.4% of its gross domestic product (GDP), equivalent to USD 19 billion. If no interventions are implemented, this figure is projected to double to 4.1% (USD 78 billion) by 2060 (3). However, the Saudi Arabian government and the Quality-of-Life Program 2020 are initiating numerous programs aimed at reducing obesity by 3% by 2030 (4).

In the past decade, several weight loss strategies, including behavioral, therapeutic, and surgical interventions, have been introduced (5). These interventions are delivered within a multidisciplinary context involving dietitians, endocrinologists, physiotherapists, and occasionally, surgeons (5). Among these, weight loss surgery (also known as bariatric or metabolic surgery) is the most effective and sustainable treatment for obesity (5, 6). Weight loss surgeries encompass procedures such as gastric bypass, sleeve gastrectomy, gastric band, and duodenal switch. These surgeries have demonstrated positive outcomes in treating conditions like diabetes, high blood pressure, sleep apnea, and high cholesterol, among others (6, 7).

Saudi Arabia has approximately 20 accredited public obesity centers that perform weight loss surgeries (8). In 2021, these centers performed over 30,000 surgeries at an average cost between USD 6,600 and 10,000, resulting in a total expenditure exceeding USD 100 million (9). These operations represent 10% of the Ministry of Health’s annual budget (3). In late 2022, the Saudi Council of Health Insurance issued a new mandate requiring all private health insurance companies to cover all types of bariatric surgery for individuals with a Body Mass Index (BMI) of 30 kg/m^2^ or above (10). The maximum coverage is USD 5,000, subject to a 20% deductible (9). In addition, in 2022, the International Federation for the Surgery of Obesity and Metabolic Disorders (IFSO) provides a global snapshot of bariatric surgery (11). According their report, 100% of bariatric procedures carried out in Saudi Arabia were sleeve gastrectomy (11).

Bariatric surgery, despite being generally superior to non-surgical interventions for weight loss, falls short of expected benefits for a significant number of patients. This shortfall primarily stems from two issues: suboptimal weight loss (SWL) and weight regain (WR) (12). SWL is identified as a failure to achieve a loss translating to 40–60% of the baseline excess body weight (EWL) over a span of 1–2 years (12). Post-bariatric surgery, approximately 11–22% of patients exhibit this suboptimal weight loss (13–18). On the other hand, the phenomenon of weight regain—defined as an initial achievement of expected weight loss post-surgery followed by a weight increase—exhibits a higher prevalence within the bariatric patient population (19, 20).

Thus, the aim of this study is to conduct a comparative analysis of lifestyle behaviors and dietary intake in obese and non-obese individuals who have undergone bariatric surgery, with the objective of understanding the variations in obesity outcomes based on these factors.

## 2. Methods

### 2.1. Data and sampling

This research is a secondary data analysis of a cross-sectional dataset retrieved from the Sharik Diet and Health National Survey (SDHNS) (21). The SDHNS, an annual nationwide cross-sectional survey conducted in Saudi Arabia, employs phone interviews. For a balanced distribution of participants, the SDHNS uses a proportional quota sampling method, stratified by age, gender, and region across Saudi Arabia’s 13 administrative regions. The SDHNS integrates ZDataCloud®, a data collection tool that minimizes sampling bias without human interaction (22). The dataset used in this research spans from 2020 to 2022, encompassing more than 15,000 participants (23). A detailed methodology of the SDHNS is available in a separate document published by the Sharik Association for Research & Studies (21). In this study, we extracted data for only people who had bariatric surgery to cover the aim of this research.

### 2.2. Measures

#### 2.2.1. Demographics

Demographic information, including age, sex, and education level was provided by the SDHNS database. Obesity was defined as a Body Mass Index (BMI) equal or above 30 kg/m^2^, calculated from self-reported height and weight.

#### 2.2.2. Self-rated health

Participants rated their health on a five-point Likert scale, ranging from 1 (poor) to 5 (excellent). Queries about bariatric surgery were made, including when the surgery took place. The response options for the duration since the surgery were: within the last 6 months or longer than 6 months. To determine the high-risk group for depression in the sample, a score exceeding 10 on the Patient Health Questionnaire-9 (PHQ-9) was used.

#### 2.2.3. Behavioral lifestyle

The participants’ level of physical activity adhered to the World Health Organization’s recommendations. It included the frequency of at least 30 min of moderate-intensity activity or 20 min of high-intensity activity within the past 2 weeks. Subsequently, the activity level was classified as moderate (150 min/week of moderate activity) or vigorous (75 min/week of vigorous activity). The activity level was deemed acceptable (ALPA) if either criterion was met or classified as low (LLPA) if not met. Sedentary lifestyle assessment was based on daily leisure hours spent sitting, divided into two categories: 4 h or less per day and more than 4 h per day.

#### 2.2.4. Dietary lifestyle

The Nutrition Guidelines for Weight Loss Surgery by Jonse Hopkins Medicine was used to assess dietary habits (24). Responses were classified according to daily consumption within each food category, then reclassified according to the guidelines. “Acceptable Level” indicates adherence to the recommendation, while “Not Acceptable Level” suggests failure to meet or exceeding the recommendation.

### 2.3. Data analysis

Descriptive data in this study were represented using counts and percentages. The bivariate analysis was conducted using the chi-square (χ^2^) test. A multivariate logistic regression model, including all variables, was conducted to further confirm the factors associated with the obesity variable. The regression model was performed twice, both with and without the “time elapsed since the surgical procedures” variable, in order to assess its potential effect on the model. Since data collection was using ZDataCloud®, no missing data occurred. SPSS version 22 was utilized for statistical analysis, and the results are reported according to the checklist for cross-sectional studies, strengthening the Reporting of Observational Studies in Epidemiology (STROBE).

## 3. Results

Out of the 15,980 participant records in the SDHNS database studied between 2020 and 2022, 806 (5.0%) had undergone bariatric surgery. These 806 eligible records were subsequently extracted and analyzed for this study. Of these 806 participants, 54.8% were female, with a mean age of 38.85 years (SD 13.02; range: 18–87), and 33.0% have obesity. Table 1 shows participant demographics characteristics classified by obesity status.

**Table 1:** Demographic characteristics of participants classified by obesity status (*n* = 806).

Variables	Had bariatric surgery			
	Not with obesity *n* (%)	With obesity *n* (%)	Total	Chi-square
Sex				
Male	247 (45.7)	117 (44.0)	364 (45.2)	0.638
Female	293 (54.3)	149 (56.0)	442 (54.8)	
Age group (in years)				
29 and less	152 (28.1)	52 (19.5)	204 (25.3)	**0.010**
30–39	161 (29.8)	75 (28.2)	236 (29.3)	
40–49	143 (26.5)	78 (29.3)	221 (27.4)	
50+	84 (15.6)	61 (22.9)	145 (18.0)	
Education				
Less than bachelor’s degree	238 (44.1)	135 (50.8)	373 (46.3)	0.074
Bachelor’s degree or above	302 (55.9)	131 (49.2)	433 (53.7)	
Time since the surgery				
6 months and less	124 (23.0)	54 (20.3)	178 (22.1)	0.392
More than 6 months	416 (77.0)	212 (79.7)	628 (77.9)	

All values in bold are significant.

Table 2 shown that self-health rate and risk of depression revealed no significant differences in health rates and depression levels between individuals with and without obesity.

**Table 2:** Self-satisfaction and risk of depression among participants classified by obesity status.

Variables	Had bariatric surgery			
	Not with obesity *n* (%)	With obesity *n* (%)	Total	Chi-square
Self-satisfaction rate				
Excellent	152 (28.1)	64 (24.1)	216 (26.8)	0.767
Very Good	179 (33.1)	89 (33.5)	268 (33.3)	
Good	120 (22.2)	65 (24.4)	185 (23.0)	
Neutral	65 (12.0)	34 (12.8)	99 (12.3)	
Poor	24 (4.4)	14 (5.3)	38 (4.7)	
Depression				
At risk	129 (23.9)	51 (19.2)	180 (22.3)	0.131
Not at risk	411 (76.1)	215 (80.8)	626 (77.7)	

Table 3 demonstrates the associations between smoking cigarettes, waterpipe usage, weight management practices, and self-weight classification with obesity.

**Table 3:** Participant lifestyle classified by obesity status.

Variables	Had bariatric surgery			
	Not with obesity *n* (%)	With obesity *n* (%)	Total	Chi-square
Physical activity				
ALPA	149 (27.6)	61 (22.9)	210 (26.1)	0.156
LLPA	391 (72.4)	205 (77.1)	596 (73.9)	
Sitting for leisure				
4 h or less/day	265 (49.1)	123 (46.2)	388 (48.1)	0.449
More than 4 h/day	275 (50.9)	143 (53.8)	418 (51.9)	
Currently smoking cigarette				
Never	391 (72.6)	212 (79.7)	604 (74.9)	**0.029**
Yes	148 (27.4)	54 (20.3)	202 (25.1)	
Currently smoking waterpipe				
Never	377 (69.8)	205 (77.1)	582 (72.2)	**0.031**
Yes	163 (30.2)	61 (22.9)	224 (27.8)	
Weight management (What are you currently doing with regard to your weight?)				
Lose more weight	190 (35.2)	207 (77.8)	397 (49.3)	**<0.001**
Keep weight as it is	197 (36.5)	28 (10.5)	225 (27.9)	
Gain more weight	38 (7.0)	5 (1.9)	43 (5.3)	
Doing nothing	115 (21.3)	26 (9.8)	141 (17.5)	
Self-weight classification				
Underweight	63 (11.7)	7 (2.6)	70 (8.7)	**<0.001**
Ideal	270 (50.0)	30 (11.3)	300 (37.2)	
Overweight	176 (32.6)	138 (51.9)	314 (39.0)	
Obese	31 (5.7)	91 (34.2)	122 (15.1)	

All values in bold are significant.

Table 4 presents the dietary intake and habitual behavior data, highlighting associations between increased consumption of chicken, fresh juice, and energy drinks among obese individuals.

**Table 4:** Dietary intake and habit of participants classified by obesity status.

Variables	Had bariatric surgery			
	Not with obesity *n* (%)	With obesity *n* (%)	Total	Chi-square
Fruit consumption				
Acceptable fruit level	49 (9.1)	22 (8.3)	71 (8.8)	0.705
Not acceptable fruit level	491 (90.9)	244 (91.2)	735 (91.2)	
Vegetable consumption				
Acceptable vegetable level	70 (13.0)	36 (13.5)	106 (13.2)	0.822
Not acceptable vegetable level	470 (87.0)	230 (86.5)	700 (86.8)	
Meat consumption				
Acceptable meat level	114 (21.1)	59 (22,2)	173 (21.5)	0.728
Not acceptable meat level	426 (78.9)	207 (77.8)	633 (78.5)	
Chicken consumption				
Acceptable chicken level	105 (19.4)	35 (13.2)	140 (17.4)	**0.027**
Not acceptable chicken level	435 (80.6)	231 (86.8)	666 (82.6)	
Seafood consumption				
Acceptable seafood level	93 (17.2)	37 (13.9)	130 (16.1)	0.229
Not acceptable seafood level	447 (82.8)	229 (86.1)	676 (83.9)	
Fresh juice consumption				
Never	139 (25.7)	104 (39.1)	243 (30.1)	**<0.001**
1–3 times/week	260 (48.1)	121 (45.5)	381 (47.3)	
4–7 times/week	141 (26.1)	41 (15.4)	182 (22.6)	
Canned juice consumption				
Never	205 (38.0)	114 (42.9)	319 (39.6)	0.287
1–3 times/week	227 (42.0)	97 (36.5)	324 (40.2)	
4–7 times/week	108 (20.0)	55 (20.7)	163 (20.2)	
Carbonated drink consumption				
Never	204 (37.8)	107 (40.2)	311 (38.6)	0.766
1–3 times/week	199 (36.9)	92 (34.6)	291 (36.1)	
4–7 times/week	137 (25.4)	67 (25.2)	204 (25.3)	
Power drink consumption				
Never	310 (57.4)	174 (65.4)	484 (60.0)	**0.026**
1–3 times/week	155 (28.7)	53 (19.9)	208 (25.8)	
4–7 times/week	75 (13.9)	39 (14.7)	114 (14.1)	
Eating food prepared outside home in the last week				
Never	141 (26.1)	79 (29.7)	220 (27.3)	0.489
1–3 meals	260 (48.1)	126 (47.4)	386 (47.9)	
4 meals and above	139 (25.7)	61 (22.9)	200 (24.8)	
Breakfast meals in the last week				
Never	74 (13.7)	45 (16.9)	119 (14.8)	0.464
1–3 meals	178 (33.0)	82 (30.8)	260 (32.3)	
4–7 meals	288 (53.3)	139 (52.3)	427 (53.0)	

All values in bold are significant.

The bi-variate chi-square analysis showed that eight variables were significantly associated with the obesity variable, including: age, cigarette smoking, waterpipe smoking, self-managed weight control, self-classification of weight, consumption of energy beverages, natural juices, and chicken consumption. However, analysis from the logistic regression model revealed that only four variables remained in the model, including: age (OR = 1.023; 95% CI = 1.011–1.036; *p* < 0.001), not acceptable chicken consumption level (OR = 1.647; 95% CI = 1.079–2.515; *p* = 0.021), current cigarette smoking (OR = 1.492; 95% CI = 1.038–2.146; *p* = 0.031), consumption of fresh juices (Never compared to 4–7 times/week; OR = 2.871; 95% CI = 1.845–4.466; *p* < 0.001), consumption of fresh juices (1–3 times/week compared to 4–7 times/week; OR = 1.754; 95% CI = 1.155–2.664; *p* = 0.008) were significantly associated with participants with obesity. However, the “time elapsed since the surgical procedures” was not statistically significant in the model and did not influence the overall results associated with the other variables.

## 4. Discussion

The goal of the study was to compare lifestyle behaviors and dietary intake between individuals who remained obese and those who did not, after undergoing bariatric surgery. The aim was to comprehend the variations in obesity outcomes due to these factors. The study revealed that bariatric surgery is quite common in Saudi Arabia, with a prevalence of 5%. Among those who underwent surgery, 33.0% still face issues with obesity. Factors associated to obesity among these individuals include age, inadequate levels of chicken consumption, low fresh juices consumption, and current smoking habits.

In terms of age, research suggests that age significantly impacts weight loss following bariatric surgery. With advancing age, the metabolic rate crucial for fat burning declines (25). This aging process can have a marked effect on the capacity for weight loss, largely due to physiological changes that occur over time (26). Notably, these changes encompass a reduction in basal metabolic rate (BMR), loss of muscle mass, and hormonal changes, all potentially contributing to increased body fat and decreased energy use (25). A decrease in BMR with age is associated with loss of muscle mass and an increase in fat mass, making it more difficult for older people to burn calories as efficiently as younger ones (27). Furthermore, age-related hormonal shifts, such as decreases in growth hormone and testosterone, can amplify muscle loss and fat gain (28). Research has revealed that people under 45 years old tend to lose more excess BMI compared to older individuals following bariatric surgery (29). This pattern may serve as a predictive marker for weight loss before undergoing bariatric surgery (29). Given these combined factors, weight loss may be more difficult for older adults, potentially requiring them to consume fewer calories and/or increase their physical activity levels more than younger individuals to achieve equivalent weight loss (30).

In the study, obese and non-obese groups consumed similar levels of meat, but the non-obese group consumed significantly more chicken. Therefore, the study findings are consistent with earlier research showing that a diet rich in protein can be linked to successful weight loss outcomes (31, 32). The study further expands on past discoveries that identified a strong connection between increased intake of chicken and red meat, decreased consumption of grains and rice, and weight loss after bariatric surgery (33). Chicken, being a lean source of high-quality protein, is crucial for creating feelings of fullness and preserving muscle mass during periods of low-calorie intake (34). After bariatric surgery, a diet high in protein and low in carbohydrates is often recommended to support weight loss and prevent weight gain (35). The observed weight loss in study participants may be associated with their shift toward a high protein diet.

Our study revealed a significant association between the consumption of fresh juice and weight reduction post-bariatric surgery. This aligns with previous research suggesting that nutrient-dense, low-calorie dietary components, such as fresh juice, could aid weight control (36). Fresh juice, abundant in essential nutrients, can serve as a valuable dietary addition that promotes a calorie deficit without compromising nutrient intake (37). Additionally, fresh juices’ high-water content can enhance feelings of fullness, potentially curbing overeating, and subsequent weight gain (38). However, it is crucial to clarify that while our study indicates an association, it does not imply causality. A multitude of factors, including physical activity, overall dietary patterns, and adherence to post-operative guidelines, heavily influence post-surgery weight management (39). Moreover, despite fresh juice’s benefits, it should not replace whole fruits and vegetables due to its typically lower fiber content (40).

In contrast to nations such as the United States, Saudi Arabia lacks a standardized nutritional guideline to be adhered to following bariatric surgery. In fact, studies conducted within the Saudi population indicates that post-operative patients exhibited a limited understanding of post-surgical nutrition, as evidenced by their performance on a post-bariatric nutritional knowledge assessment. This is noteworthy considering a significant proportion of these patients held at least an undergraduate degree. One plausible explanation for this knowledge gap might be rooted in the prevailing practices of the Saudi healthcare system. Specifically, patients typically only receive dietary consultations after the scheduling of their bariatric procedure. Such a delay might be attributed to the absence of a structured educational framework, prolonged wait times, or challenges related to patients maintaining their scheduled clinic visits.

The reported incidence of smoking among patients who have undergone bariatric surgery can reach up to 40% (41). A systematic review encompassing 48 studies revealed that smoking has minimal or no impact on weight loss subsequent to bariatric surgery (41). Nonetheless, the effect documented in the current study appears to be associated with the healthier lifestyle adopted by the non-obese group. This includes habits such as consuming a high protein diet and fresh juices, and a lower prevalence of smoking. For those considering bariatric surgery, it is often recommended to quit smoking (42). This co-occurrence potentially amplifies the indication that non-smokers generally maintain a healthier lifestyle.

This study provided an exploration of both lifestyle and eating habits. However, a more thorough probe into the variables discussed herein is necessary. Subsequent studies should aim to enrich our understanding by collecting more comprehensive data from qualitative and longitudinal research, to better differentiate between these two cohorts post-bariatric surgery.

This study has several limitations that should be noted. First, the cross-sectional design, although allowing for a broad analysis, is unable to establish cause-and-effect relationships between two post bariatric participants and the psychological and behavioral outcomes measured. Because we were assessing participants at a single point in time, we could not definitively determine whether the surgery led to these outcomes or if these conditions were pre-existing. Second, our research was conducted using a secondary data analysis. While this method is efficient and allows for larger sample sizes, it limits the type of data that can be explored. We were constrained by the variables and measures that were already included in the existing dataset, which may not have included all potentially relevant factors. In terms of generalizability, our study sample may not be representative of the entire population of individuals who have undergone bariatric surgery. Factors such as age, sex, ethnicity, comorbidities, and other sociodemographic variables can all impact mental health and eating behavior outcomes. An additional limitation of this study is the utilization of self-reported data. The inherent nature of self-reporting might result in the overestimation or underestimation of behavior by a factor of three to four times the actual occurrence within certain contexts. Future studies should consider incorporating these factors to provide a richer, more nuanced understanding of the experiences of bariatric surgery patients. These limitations highlight the need for further research, particularly longitudinal studies, to verify our findings and to further investigate the intricate relationship between bariatric surgery, mental health, and eating behaviors.

## 5. Conclusion

The study results underscore the nuanced interplay between lifestyle factors and obesity outcomes post-bariatric surgery. Findings reveal that among patients who underwent bariatric surgery, a considerable proportion (33.0%) continue to grapple with obesity. Various factors were associated with persistent obesity, including advanced age, lower consumption of chicken and fresh juices, and current smoking practices. While the research unravels key connections, it should be interpreted cautiously as it does not establish causality. The identified factors could inform pre- and post-operative care protocols to enhance bariatric surgery outcomes. Further prospective and interventional studies are recommended to confirm these findings, investigate causality, and explore potential interventions, such as diet adjustments and smoking cessation support.

## Data availability statement

The raw data supporting the conclusions of this article will be made available by the authors, without undue reservation.

## Ethics statement

The studies involving humans were approved by Sharik Association for research and studies. The studies were conducted in accordance with the local legislation and institutional requirements. The participants provided their written informed consent to participate in this study.

## Author contributions

NAA: Formal analysis, Investigation, Methodology, Writing – original draft. NB: Supervision, Writing – review & editing. AEA: Supervision, Writing – review & editing. NA: Data curation, Writing – review & editing. RA: Data curation, Writing – review & editing. AA: Supervision, Writing – review & editing.
